# Zika Virus in a Traveler Returning to China from Caracas, Venezuela, February 2016

**DOI:** 10.3201/eid2206.160273

**Published:** 2016-06

**Authors:** Jiandong Li, Ying Xiong, Wei Wu, Xiaoqing Liu, Jing Qu, Xiang Zhao, Shuo Zhang, Jianhua Li, Weihong Li, Yong Liao, Tian Gong, Lijing Wang, Yong Shi, Yanfeng Xiong, Daxin Ni, Qun Li, Mifang Liang, Guoliang Hu, Dexin Li

**Affiliations:** National Institute for Viral Disease Control and Prevention, Chinese Center for Disease Control and Prevention, Beijing, China (Jiandong Li, W. Wu, J. Qu, X. Zhao, S. Zhang, L. Wang, M. Liang, D. Li);; Jiangxi Provincial Center for Disease Control and Prevention, Nanchang, China (Y. Xiong, X. Liu, T. Gong, Y. Shi, G. Hu);; Ganzhou Municipal Center for Disease Control and Prevention, Ganzhou, China (Jianhua Li, Y. Liao, Y. Xiong);; Office of Emergence Response, Chinese Center for Disease Control and Prevention, Beijing (D. Ni, Q. Li);; Beijing Center for Disease Prevention and Control, Beijing (W. Li)

**Keywords:** Zika virus, ZIKAV, Jiangxi, China, flavivirus, Caracas, Venezuela, vector-borne infections, viruses, travel, Aedes, Aedes aegypti, Aedes albopictus, mosquitoes

**To the Editor:** Zika virus, a member of the *Flaviviridae* family, is primarily transmitted through *Aedes* spp. mosquitoes, and evidence of vertical, sexual, and blood transmission of Zika virus has been reported (*1*–*3*). The virus has spread rapidly across Latin America and the Caribbean since the end of 2014 and has been linked to an increase in neurologic disorders and neonatal malformations in these areas (*4*). Zika virus has the potential to spread internationally through the carriage of goods and travelers (*5*). Traveler volume between China and areas with autochthonous transmission of Zika virus is increasing; in 2015, China received ≈84,000 travelers who had departed from international airports in Brazil (*5*). The *Ae. aegypti* mosquito, the competent vector for Zika virus, is found in areas of Hainan, Guangdong, and Yunnan provinces on the mainland of China, where the known distribution is limited to areas below 22° latitude. However, *Ae. albopictus* mosquitoes are widely distributed, extending from the southern reaches to the northern and western parts of China, with north fringes from Shenyang in Liaoning Province, through Tianshui and Longnan in Gansu Province, to Motuo in Tibet (*6*). Surveillance of Zika virus infection among Chinese travelers has been enhanced since January 2016. We report the clinical and laboratory findings for a case Zika virus infection imported from Venezuela.

A previously healthy 34-year-old Chinese man was admitted to the Hospital of Ganxian on February 6, 2016. He had worked in Caracas, Venezuela, during January 1–February 2 and had onset of fever (38.0°C), headache, and dizziness on January 28. He subsequently had rash, chills, retro-orbital pain, and mild diarrhea on February 2, the day on which he departed from Caracas and traveled to Jiangxi Province via Paris and Hong Kong, arriving in Shenzhen, China, on February 5. At the time of hospital admission, the patient had fever (36.7°C), headache, conjunctivae, rash on his back and face, retro-orbital pain, and mild diarrhea. General clinical examination was unremarkable. Results of a complete blood cell count and liver function tests were within reference ranges.

Serum samples were collected at day 9 and day 10 after symptom onset, and urine samples were collected once a day from day 10 through day 14. In the 2 serum samples, no dengue virus (DENV) or chikungunya virus (CHIKV) IgM or IgG were detected by a Panbio IgM and IgG capture ELISA for DENV (Panbio, Queensland, Australia) or by an indirect immunofluorescence assay slide test kit for CHIKV (EUROIMMUN AG, Lübeck, Germany). Serum and urine samples were negative for DENV nonstructural protein 1 (NS1) antigen on an NS1-ELISA test kit (Wantai Bio-Pharm, Beijing, China). To detect virus RNA in samples, in-house–designed probe and primers specific to DENV and CHIKV were used (*7*). The PCR for Zika virus was targeted to the NS1 gene. RNA was extracted from 140 μL of serum or urine by using the QIAamp Viral RNA Mini Kit (QIAGE, Hilden, Germany). Amplification reactions were performed by using the AgPath-ID One-Step RT-PCR Kit (Ambion, Carlsbad, CA, USA). A standard curve with serial dilutions of known concentrations of in vitro–transcribed RNA from a reference plasmid was used to estimate viral load in samples. Test results for DENV and CHIKV were negative. However, the serum sample collected at day 9 was positive for Zika virus RNA (viral load 1.4 × 10^4^ copies/mL), and Zika virus RNA was detected from urine samples collected on days 10, 11, and 12 (viral loads 8.6 × 10^4^, 4.5 × 10^4^, and 1.2 × 10^4^ copies/mL, respectively).

Next-generation genomic sequencing of the Zika virus genome was conducted by using the MiSeq platform (Illumina, Hayward, CA, USA) on serum and urine samples. A 1,813-bp of partial genome sequences (strain VE_Ganxian, GenBank accession no. KU740199) was obtained from urine and was used for comparing with selected other strains from GenBank. Phylogenetic analysis showed that the virus was of Asian lineage (Figure). Pairwise genetic distance calculation indicated that the sequence was most closely related to other viruses reported from French Polynesia in 2013 (strain H/PF/2013), the Caribbean in 2014 (strain Haiti/1225/2014), and Latin America in 2015 (strain ZikaSPH2015), having a 99.4% similarity in sequence.

**Figure:**
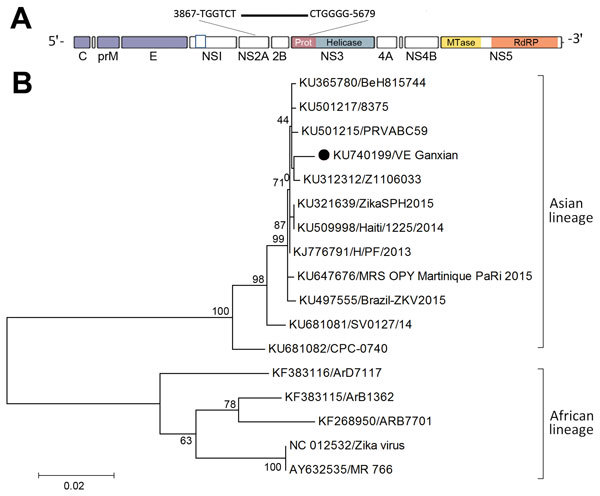
Phylogenetic analysis of partial sequences of Zika virus for an imported case of Zika virus infection in a traveler returning to China from Caracas, Venezuela, February 2016, compared with selected other strains from GenBank. A) Schematic diagram showing the contiguous sequence, obtained from de novo assembly and BLAST (http://blast.ncbi.nlm.nih.gov/Blast.cgi), targeted at the 3′ terminus of nonstructural protein 2B and the 5′ terminus of nonstructural protein 3 genes (figure not drawn to scale). B) Maximum-likelihood phylogenetic tree inferred based on the Tamura-Nei model (*8*). The partial sequence of VE_Ganxian (black dot) obtained in this study was analyzed against 11 reference strains of Asian lineage and 5 reference strains of African lineage. All positions containing gaps and missing data were eliminated. Evolutionary analyses were conducted in MEGA version 7.0 (http://www.megasoftware.net). GenBank accession numbers are given. Scale bar indicates number of substitutions per site. C, capsid; E, envelope; MTase, methyltransferase; PrM, premembrane; RdRP, RNA-dependent RNA polymerase.

The clinical findings for the patient were similar to those previously reported among Zika virus–infected patients (*9*), although no arthralgia was apparent. Viral RNA remained detectable for 9 days after symptom onset in serum and for an additional 3 days in urine. We did not test this patient’s semen and thus cannot comment on risk for onward sexual transmission; however, the patient was told about the risks for sexual transmission of Zika virus and was advised to adopt safer sexual practices or to abstain from sexual activity for at least 1 month after recovery. In February, the mosquito density is low in Jiangxi Province (*10*), suggesting that this imported case is unlikely to cause mosquitoborne transmission. However, with the onset of summer and increased density of *Aedes* mosquitos, the risk for onward transmission of travel-associated Zika virus should not be overlooked.
